# Variation in the concentration and regional distribution of magnetic nanoparticles in human brains, with and without Alzheimer’s disease, from the UK

**DOI:** 10.1038/s41598-021-88725-3

**Published:** 2021-04-30

**Authors:** Jessica Hammond, Barbara A. Maher, Imad A. M. Ahmed, David Allsop

**Affiliations:** 1 Division of Biomedical and Life Sciences, Faculty of Health and Medicine, Lancaster University, Lancaster, LA1 4YQ UK; 2 Centre for Environmental Magnetism and Palaeomagnetism, Lancaster Environment Centre, Lancaster University, Lancaster, LA1 4YQ UK; 3 Department of Earth Sciences, University of Oxford, Oxford, OX1 3AN UK

**Keywords:** Neuroscience, Alzheimer's disease, Magnetic properties and materials, Nanotoxicology

## Abstract

The presence of magnetic nanoparticles (MNPs) in the human brain was attributed until recently to endogenous formation; associated with a putative navigational sense, or with pathological mishandling of brain iron within senile plaques. Conversely, an exogenous, high-temperature source of brain MNPs has been newly identified, based on their variable sizes/concentrations, rounded shapes/surface crystallites, and co-association with non-physiological metals (e.g., platinum, cobalt). Here, we examined the concentration and regional distribution of brain magnetite/maghemite, by magnetic remanence measurements of 147 samples of fresh/frozen tissues, from Alzheimer’s disease (AD) and pathologically-unremarkable brains (80–98 years at death) from the Manchester Brain Bank (MBB), UK. The magnetite/maghemite concentrations varied between individual cases, and different brain regions, with no significant difference between the AD and non-AD cases. Similarly, all the elderly MBB brains contain varying concentrations of non-physiological metals (e.g. lead, cerium), suggesting universal incursion of environmentally-sourced particles, likely across the geriatric blood–brain barrier (BBB). Cerebellar Manchester samples contained significantly lower (~ 9×) ferrimagnetic content compared with those from a young (29 years ave.), neurologically-damaged Mexico City cohort. Investigation of younger, variably-exposed cohorts, prior to loss of BBB integrity, seems essential to understand early brain impacts of exposure to exogenous magnetite/maghemite and other metal-rich pollution particles.

## Introduction

Iron is an essential and versatile element present in various forms throughout the brain, and its most abundant transition metal. Its ubiquity reflects its ease in changing solubility via valence state; gaining an electron to convert from insoluble ferric (Fe^3+^) to soluble ferrous state (Fe^2+^) and vice versa. Iron not only participates in biochemical reactions, such as the generation of ATP in mitochondria and transport of oxygen via haemoglobin, but is also a crucial part of brain-specific functions such as axon myelination^1^ and synthesis of various neurotransmitters, like dopamine^2^. This versatility comes at a price; the ease of valence change can have detrimental effects if, for example, free (unbound) iron becomes available to react with oxygen and produce damaging free radicals. To protect the body from the ‘darker’ side of iron reactivity, iron levels and compounds are tightly regulated. Excess labile (free) iron is sequestered and stored, and ferrous iron oxidised to less reactive ferric forms.

Regional accumulation of iron and of magnetite in the brain during normal ageing has been reported^3–5^ but is also evident in several neurodegenerative diseases (NDD) including Alzheimer’s disease (AD)^6^, Parkinson’s disease (PD)^7^ and amyotrophic lateral sclerosis (ALS)^8^. Meta-analysis of published data (up to 2014) indicates elevated iron in several regions of AD brains: the caudate nucleus; globus pallidus; cingulate cortex; putamen; amygdala; temporal lobe; parietal lobe; and frontal lobe^9^. A common theme amongst NDD is evidence of oxidative damage and dyshomeostasis of metals including iron, copper and zinc^10–12^. Iron (particularly Fe^2+^) can cause oxidative damage (stress) by generating reactive oxygen species (ROS) via the Fenton reaction^13^. ROS, including superoxide anion (O_2_^−^), peroxide (O_2_^2−^) and hydroxyl radicals (HO^·^), are highly reactive, interacting indiscriminately with different cellular components including DNA, lipids, and proteins leading to altered enzyme activity, mutations and membrane permeability. These interactions may ultimately lead to cell death via apoptosis, autophagy, necrosis or ferroptosis^14^.

To protect the brain from ROS-induced damage, iron is tightly regulated by two key proteins: the iron transporter protein, transferrin; and the iron storage protein, ferritin. Ferritin comprises a protein cage, approximately 12 nm in diameter, with an 8 nm hollow core. The protein cage contains threefold and fourfold ion transporter channels that facilitate iron transport into the core, which can hold up to 4500 atoms of iron^15^. Fe^2+^ is oxidised within this core to the less toxic Fe^3+^ and stored as ferrihydrite (Fe_2_O_3_·0.5(H_2_O)). Interruptions to this process may be pathological; reduced iron capacity of transferrin^16^, impaired functioning of ferritin^17^ or decreased expression of ferritin^18^ are all proposed mechanisms for the iron accumulation observed in NDD^19^.

Another suspected cause of reportedly increased iron in the ageing brain is the presence of discrete particles of iron oxides, including magnetite^20–22^, maghemite^20–22^, ferrihydrite^23^, goethite^23^, haematite^22,24^ and wüstite^23^. Of these iron oxides, magnetite may be of particular importance due to its Fe^2+^ content (Fe^2+^ can participate in Fenton chemistry), and its reported direct associations with amyloid plaques^25,26^ and NDD^27^. Magnetite (Fe_3_O_4_) is a strongly magnetic material, comprising a close-packed array of oxide (O^2−^) ions; Fe^3+^ ions occupy tetrahedral holes and both Fe^3+^ and Fe^2+^ occupy octahedral holes. Magnetite nanoparticles often display some degree of surface oxidation towards their oxic counterpart, maghemite^21^.

Three different forms of magnetite/maghemite nanoparticles (MNPs) have been reported to occur in the human brain: biogenic MNPs (with a suggested role in magnetic field sensing); plaque-associated MNPs (reportedly arising from iron mishandling); and exogenous MNPs (displaying high-temperature morphologies and co-associated with other metals, including non-physiological species). Those biogenic MNPs which have been linked with sensing of the Earth’s magnetic field are well-formed, euhedral particles (i.e., with sharp, well-defined crystal faces), and mostly around 10–70 nm^20^. Such well-formed MNPs strongly resemble MNPs formed in other organisms, such as magnetotactic bacteria; their ‘bio-engineered’ size optimises the torque sensed from the geomagnetic field^20^. In magnetotactic bacteria, formation of such well-formed magnetite crystals, with optimised particle sizes, occurs through biomineralization of magnetite, possibly via a ferrihydrite precursor, within membrane-bound organelles (‘magnetosomes’)^28^.

More recently, in contrast to these euhedral endogenous MNPs, dominantly-rounded MNPs have been observed directly associated with senile plaques, key pathological hallmarks of AD^26,29^. These plaque-associated MNPs were 8 to ~ 80 nm in diameter, co-localised with β-amyloid (Aβ) fibres, and comprised a mixture of magnetite and maghemite (the oxidised counterpart of magnetite), with Fe^2+^ and zero-valent iron also present within the plaque cores^26,29^. MNPs in the 2–7 nm range were also reported, consistent with formation inside the ferritin core^26^. Conversely, those plaque-associated MNPs of several 10s of nm^26^ greatly exceed ferritin core dimensions. Plascencia-Villa et al. thus suggest a two-stage formation process; smaller MNPs formed via in situ transformation of ferrihydrite within ferritin cores, which then somehow agglomerate to form the much larger, often rounded magnetite particles observed, up to ~ 80 nm diameter^26^ and sometimes up to 200–600 nm^29^. A role for Aβ in the biosynthesis of MNPs has also been proposed. Isolated amyloid plaque cores from AD patients were found to contain iron in a range of oxidation states; and Aβ suggested to cycle iron from ferrihydrite to magnetite to wüstite, resulting in generation of a constant stream of ROS-producing iron species^29^. Hence, these plaque-associated magnetite and co-occurring iron oxide particles are suggested to be endogenous, resulting from iron dyshomeostasis related to NDD^29^. The proposed precipitation of pathological MNPs in human brain ferritin^23,24^ might reflect failure to complete Fe^2+^ oxidation, or an overload of ferritin, possibly accounting for the elevated iron levels observed in AD^27^.

Additionally, or alternatively, inhalation of exogenous, airborne magnetite/maghemite pollution particles can constitute another source of rounded brain MNPs, larger than those formed within ferritin cores^21,30^. The characteristic morphology (e.g. often rounded shapes and interlocking surface crystallites), and size distribution (<10 to > 100 nm) of MNPs found in the frontal cortex, and their association with other metals (particularly metals not normally present in the body, such as platinum) mirror those of the MNPs which occur in abundance in airborne particulate matter (PM) pollution^21^—at heavily-trafficked roadsides, for example. Similarly distinctive MNPs and co-associated metals have been found in the human brainstem^31^, heart^32,33^, blood and pleural effusions^34^ and placenta^35^. Magnetite and other iron-rich NPs are formed readily at high temperatures (i.e. > 100 °C) as by-products of combustion and friction^36,37^, occurring at particle number concentrations of ~ 10^8^/m^3^ air at the roadside, for example^21^. Other prolific sources of airborne MNPs include coal-burning power plants, industrial sources, and indoor emissions, e.g., from open fires^38^ and office printers^39^.

The discovery of intact air pollution nanoparticles inside the frontal cortex indicates inhalation as their portal of entry, followed by translocation via the olfactory bulb^21,40^. In animal studies with male Fischer-344 rats exposed to high doses of titanium dioxide, nanoparticles < 200 nm in diameter accessed the olfactory bulb directly^40^, evading the blood brain barrier (BBB). The presence of distinctive, acicular titanium-rich nanoparticles both in the brainstem and the neuroenteric system indicates ingestion/swallowing of environmental particles as an additional nanoparticle portal of entry^31^.

MNPs have been the subject of numerous toxicity studies, with mixed results e.g.^41,42^. Given the association of MNPs with senile plaques^25,26,29^, peroxidase-like activity^43^, enhancement of Aβ aggregation^44^ and reported ability to disrupt microtubule dynamics (perhaps by binding to the microtubule protein tau)^45^, exposure to airborne MNPs has been identified as a potential environmental risk factor for NDD^21,30^. Exposure to airborne particulate matter (PM) is significantly associated with increased incidence of NDD. In the U.S., the risk of dementia in older women almost doubled in places where exposure to PM_2.5_ (aerodynamic diameter < 2.5 µm) was greater than the Environmental Protection Agency’s standard. A large-population (2.2 million) study in Ontario, Canada found that residents living within 50 m of major roads had up to 14% increased prevalence of dementia^46^. Although it is not yet clear which specific component/s of PM are causally related to this increased risk, it seems improbable that repeated inhalation of exogenous Fe^2+^-bearing and other iron-rich nanoparticles, and their uptake (and possible dissolution) within the brain, constitutes a harmless exposure^30^.

In counter-argument, a recent study of seven whole brains, divided into cerebral cortex, cerebellum and brainstem (not from AD cases), reported a uniform distribution of magnetite/maghemite between each brain; highest concentrations localised in the brainstem, followed by the cerebellum and the cerebral cortex^47^. These authors suggest that their observed uniformity of magnetite/maghemite distribution indicates a genetic, rather than environmental, control; and that the olfactory bulb pathway can be discounted as a potential entry portal for MNPs found distally within the brainstem. However, the brain samples in this study had been stored for several decades in formalin; some evidence exists for magnetite dissolution in tissues exposed to formalin^48^.

Given that brain iron appears to be locally elevated in various NDDs, knowledge of the distribution and forms of iron found in disease and non-disease states is important for understanding whether iron perturbation is causally linked to NDD. The first identification of magnetite/maghemite in human brains was made using superconducting quantum interference device (SQUID) magnetometry^20^, a powerful tool for identifying the location and concentrations of magnetically-ordered iron in the brain. Few studies so far have compared the concentration of MNPs in AD and control brains. Kirschvink et al. found no difference between 4 normal and 2 AD brains (samples comprising cerebral cortex and cerebellum)^20^; Bulk et al. saw no difference between 22 AD and 14 control samples (temporal cortex)^49^. A recent study reported no difference in magnetite concentration (but higher ferrihydrite concentrations) in the medial temporal gyrus of 9 AD patients compared to 9 controls^50^. In contrast, when looking at females specifically, higher magnetite concentrations were reported in the superior temporal gyrus of AD patients compared to controls (n=3 AD and 3 controls, with a subsequent study combining those samples with 16 new samples)^51^.

Here, we used SQUID magnetometry to examine the concentration of magnetite/maghemite in AD cases and age-matched controls (19 AD cases, 11 controls, aged 80-98 years from the Manchester Brain Bank, UK), across five brain regions: the frontal, temporal and occipital lobes; the cerebellum; and the entorhinal cortex. This is the first investigation, to our knowledge, of regional ferrimagnetic variations in AD cases. Additionally, we examined the concentration of some non-physiological, exogenous (i.e., pollution-derived) metals—specifically, lead, cerium, platinum and aluminium—in each brain region, in both AD and non-AD samples.

Our new data identify marked variations in the concentration and distribution of magnetite/maghemite throughout the different brain regions. Significantly higher ferrimagnetic concentrations were found in the frontal lobe compared to the entorhinal cortex. The highest magnetite/maghemite concentration of all Manchester samples analysed was found in the frontal cortex of a 93-year-old female AD case. However, no significant difference in ferrimagnetic concentration was found between our elderly AD and control cases. The Manchester brain magnetite/maghemite concentrations were on average 11× higher than those reported for formalin-stored brains. Cerebellar magnetic concentrations for the Manchester cases were ~9× lower than those measured by us for samples from a much younger, neurologically-damaged cohort, from the more highly-polluted Mexico City area^31^. Unequivocally exogenous metal species (lead, cerium, platinum, aluminium) were found in similar concentrations in both AD and control Manchester cases.

## Methods

### Brain samples

Tissue samples were supplied by the Manchester Brain Bank (MBB), U.K. (part of the Brains for Dementia Research programme, jointly funded by Alzheimer’s Research UK and the Alzheimer’s Society) with ethical review and approval by the MBB Management Committee and the Newcastle and North Tyneside I Regional Ethics Committee. Institutional ethics approval was granted by the Faculty of Health and Medicine Research and Ethics Committee (Lancaster University). All samples were obtained, processed, and stored in accordance with the Human Tissue Act and institutional ethical guidelines. Individuals (or their relatives) elect (with informed consent) to donate to the MBB, producing a self-selecting, elderly study group. A large (~ 30 g) sample of fresh/frozen tissue was obtained from the cerebellum, and frontal, occipital, and temporal lobes, from 30 individual cases (Supplementary Table S2). Of the 30 cases, 17 were female (14 AD, 3 controls) and 13 male (5 AD, 8 controls), age range 80 to 98 years. For 27 of these cases, the entorhinal cortex was sampled as a separate entity (~ 0.5 g), whereas the ‘temporal lobe’ samples may or may not encompass entorhinal cortex tissue. The entorhinal cortex was chosen as a region of interest as it is a site of early AD pathology^52^. All samples originated from the right hemisphere; accompanying data including Braak staging, plaque, and tangle counts were from the corresponding left hemisphere sample (provided by the MBB). Cases were selected to give broadly a group of cases that were cognitively normal (controls, n = 11) and a group with AD/dementia pathology (n = 19) ranging from Braak stage III to VI^53^. Cases were classified as either control or AD based on their pathology (Supplementary Table S2).

### Subsampling and control for airborne contamination

The MBB samples were obtained and frozen between 6 and 156 h post-mortem. Care was taken to ensure minimum exposure of samples to potential sources of magnetic contamination, and any such source was quantified (Supplementary Table S1). Sample external surfaces were trimmed with a ceramic knife to remove any contamination incurred during autopsy (e.g. metal fragments from surgical instruments or the saw used to open the skull). Each trimmed sample was divided into 3 approximately equal sub-samples, with an additional small subsample (0.2 g) taken for metals analysis by ICP-MS. All sub-sampling and processing of samples took place in a class II biological safety cabinet inside a class III biological laboratory. Surfaces and the ceramic knife were treated with 70 % ethanol (with unmeasurable magnetic content). The magnetic content of the safety cabinet air throughflow was quantified, by saturation isothermal remanent magnetisation (SIRM) measurements of pumped air samples, on magnetically-‘clean’ polytetrafluoroethylene (PTFE) filters.

The tissue samples were freeze-dried for 48 h using a Christ Alpha 2–4 LD plus freeze drier. Freeze-dried masses range from 0.125 to 3.054 g (average 0.781 g), except for the much smaller entorhinal cortex samples (average 0.080 g). To preclude operator bias, samples were prepared, measured and analysed blind. All magnetic values were mass-normalized for freeze-dried brain weight (kg).

### Magnetic analyses

The SIRM can be used to approximate ferrimagnetic concentrations^21^. All measurements were carried out at room temperature (293 K ± 0.5 K) using SQUID magnetometry, with a RAPID 2G DC magnetometer (2G Enterprises, California USA; mean background noise level ~ 1 × 10^−12^ Am^2^), at the Centre for Environmental Magnetism and Palaeomagnetism, Lancaster University. The natural remanent magnetisation (NRM; i.e., the sample magnetisation prior to artificial magnetisation in the lab) and/or SIRM of each individual styrene sample pot (and clingfilm) was measured and subtracted, in order to isolate the NRM and/or SIRM of the tissue sample. Sample NRMs were measured for the majority of Manchester samples (83 of 147 samples) (Supplementary Fig. S1). No a priori assumptions were made with regards to sample NRM values; measurable NRMs might result from viscous remanent magnetisation (VRM) acquired from prior, in situ magnetisation of tissue (e.g. via environmental magnetic fields). Based on typical ‘background’ readings (holder, pot, clingfilm), we determined an experimental cut-off value of 2 × 10^−11^ Am^2^; any values below this were deemed unmeasurable. Similarly, van der Weerd et al. report their noise floor as of the order of 10^−11^ Am^2^^50^. On the basis of our cut-off value, 5 Manchester samples were unmeasurable (for SIRM).

SIRMs were generated in a direct (DC) field of 1 Tesla (T), using a Newport 4” Electromagnet Type A; demagnetisation steps of 5, 10, 15, 20, 25, 30, 40, 50, 75 and 100 milliTesla (mT) utilised the 2G’s integrated AF demagnetiser. IRM acquisition curves were obtained for a sub-set of samples, via stepwise DC fields of 10, 20, 50, 100, 200, 300 and 1000 mT using the 2G IRM unit. Samples were measured immediately following acquisition of SIRM. Concentrations of magnetite (and maghemite) were calculated from the measured SIRM values, using experimentally-derived values of 13.8 Am^2^ kg^−1^ for ~ 31 nm interacting, mixed, single domain and superparamagnetic magnetite^54^ (and 12.0 Am^2^ kg^−1^ for ~ 31 nm interacting, mixed, single domain and superparamagnetic maghemite (Maher, unpublished data)).

### Metals analysis

A small (0.2 g) fresh frozen subsample was taken from each sample (not from the entorhinal cortex due to the limited mass available). Samples were accurately weighed (wet-weight) in acid-cleaned PTFE containers and digested at 60 °C for 6 h in a digestion solution buffered at pH 7.0. The digestion solution comprised 20 units/mL purified papain (Sigma Aldrich), 0.5 mM EDTA and 0.5 mM cysteine. All solutions and reagents were prepared from MillliQ water (≥ 18.2 MΩ) in a metal-free laboratory. The digestion solution was then filtered using (< 0.1 μm PTFE Omnipore membrane filter) to preclude any particulate contamination from the papain. At the end of this enzymatic digestion, solutions were transferred to 50 ml acid-washed Teflon centrifuge tubes and centrifuged at 4500 rpm for 45 min. The clear supernatant was then filtered (< 0.1 μm PTFE membrane filter) and acidified using 2% ultrapure HNO_3_ (Optima, Fisher Scientific) for bioavailable trace metals analysis using a Perkin Elmer NexION 350D inductively coupled plasma mass spectrometer (ICP-MS) with a reaction/collision cell. Helium was used as a collision gas and ammonia used in the dynamic reaction cell to eliminate polyatomic interferences. The instrument was calibrated with 31 elements of a working standard and verified by SLRS-6 water Certified Reference Material.

### Statistical analysis

All statistical analysis was conducted using SPSS 24 software (IBM). Normality tests were performed using the Shapiro–Wilk test. Data were log-transformed to reduce skewness. Specific tests were either a one-way ANOVA (with Tukey’s post-hoc), Kruskal–Wallis test, independent samples t test or independent samples Mann–Whitney U test, depending on the degree of normality of the data. Significance level (α) was set to 0.05.

## Results

**Figure 1:**
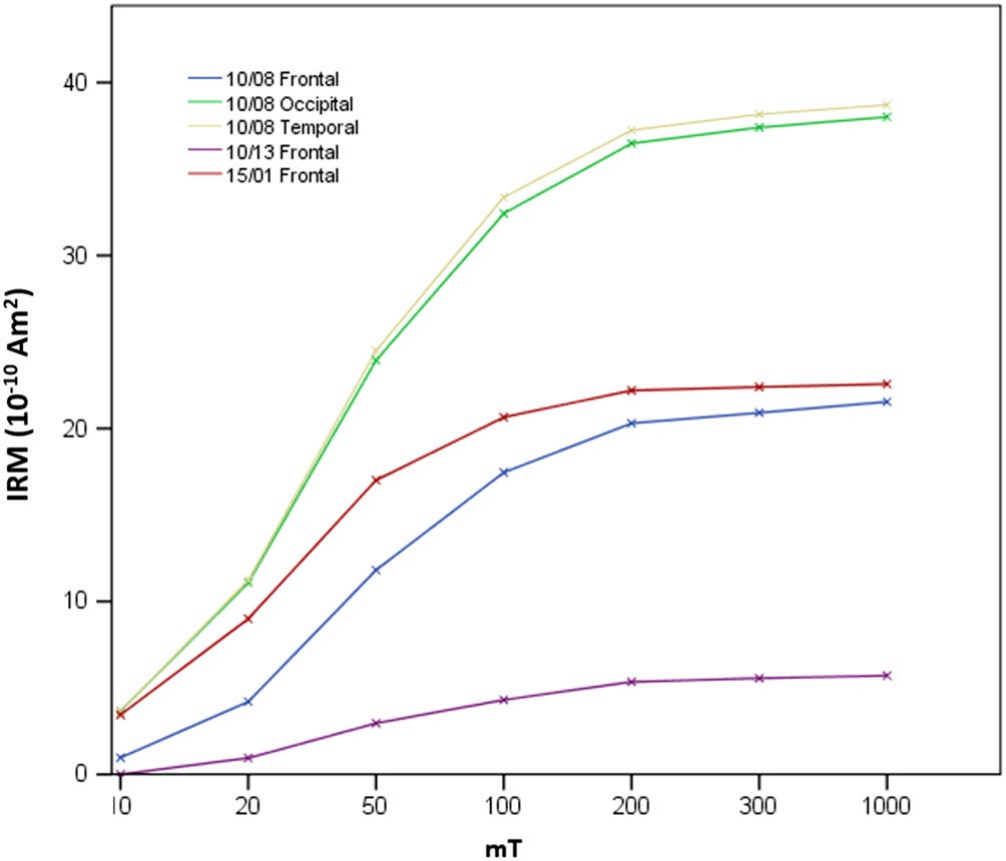
Room-temperature IRM acquisition of human brain tissue, Manchester Brain Bank samples.

Measurable amounts of ferrimagnetic material (as determined by SIRM) were detected in most of the Manchester samples (141 measurable; 6 unmeasurable). As for previous brain samples from Manchester, and Mexico City^21^, the majority of magnetic remanence is acquired by 100 mT (Fig. 1), and close to saturation at 300 mT. Such behaviour is typical of the presence of a magnetically-‘soft’, magnetite-like component, and consistent with other studies of magnetite/maghemite in human brain tissue^20,21^.

**Figure 2:**
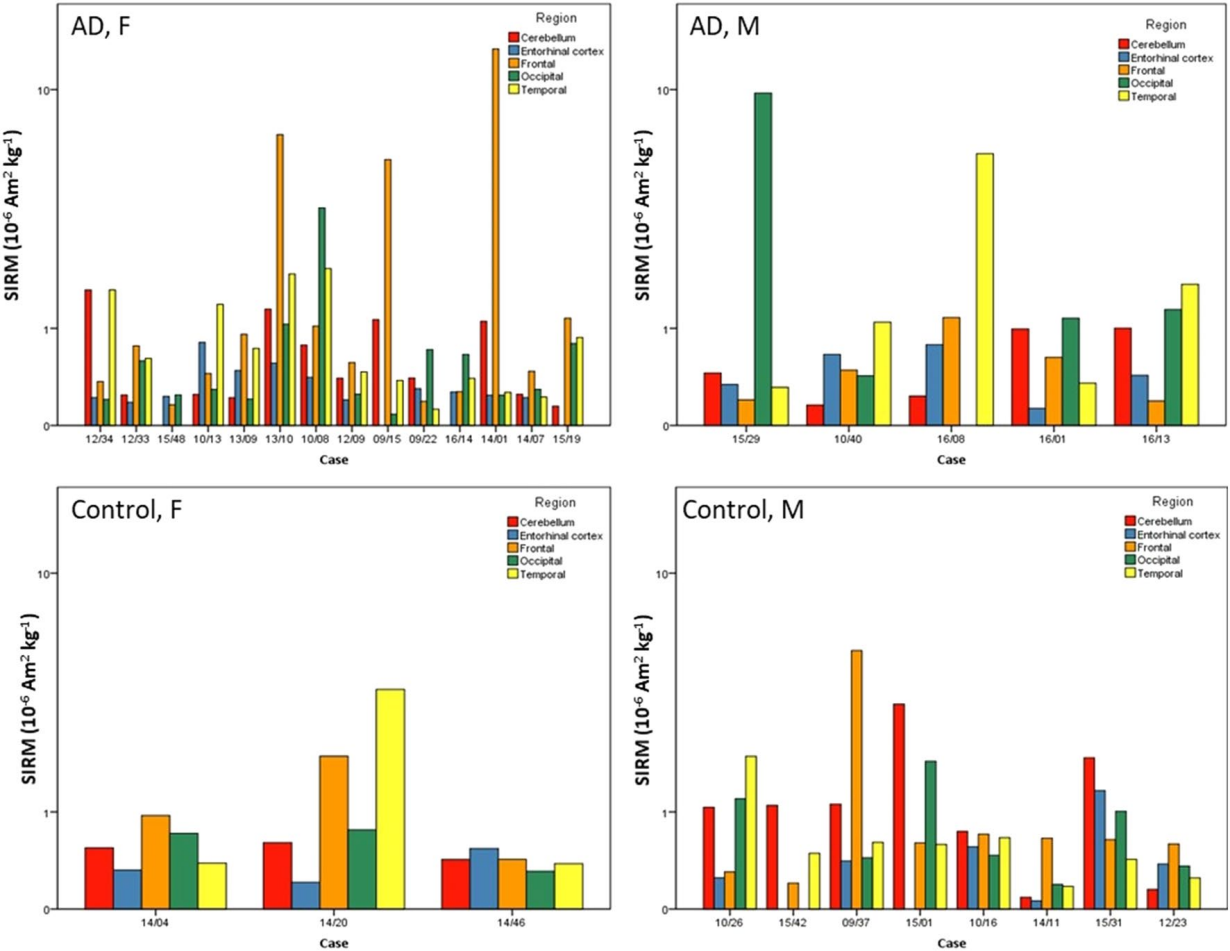
Regional distribution of mass-normalised SIRM values for human Alzheimer’s disease (AD) and control brains from Manchester Brain Bank, UK. Cases are arranged in each panel by ascending age (80–98 years) and split by sex (F = female, M = male).

Mass-normalized SIRMs of the Manchester brain samples ranged from 0.06 to 13.69 × 10^−6^ Am^2^ kg^−1^ (median 0.49 × 10^−6^ Am^2^ kg^−1^), equating to between ~ 4 and 992 ng of magnetite g^−1^ tissue (median 36 ng magnetite g^−1^) and median of 0.44 × 10^9^ MNPs g^−1^ (or between ~ 5 and 1140 ng of maghemite g^−1^ tissue (median 41 ng maghemite g^−1^) and median of 0.53 x 10^9^ MNPs g^−1^ (Table 1, Figure 2). These SIRM values are on average ~11× higher than those reported for formaldehyde-stored brains (at 293 K)^47^. They are similar in magnitude (Supplementary Fig. S4) to those obtained (at 5 K and 100 K) for fresh/frozen brain samples, from the medial temporal gyrus^50^ (SIRMs measured at temperatures below 293 K e.g. at 77 K or 100 K, are often higher, as they capture the additional remanence of magnetic particles so small (< ~ 30 nm) as to be magnetically unstable at room temperature).

**Table 1:** Magnetite mass and particle number concentrations for human brain samples from AD and control brains from MBB, UK.

	AD	Control
Min SIRM (10^–6^ Am^2^ kg^−1^)	0.08	0.06
Max SIRM (10^–6^ Am^2^ kg^−1^)	13.69	5.33
Median SIRM (10^–6^ Am^2^ kg^−1^)	0.45	0.56
Min magnetite ng g^−1^	5.79	4.35
Max magnetite ng g^−1^	992.03	386.23
Median magnetite ng g^−1^	32.68	40.58
Min particles 10^9^ g^−1^	0.07	0.05
Max particles 10^9^ g^−1^	12.29	4.78
Median particles 10^9^ g^−1^	0.40	0.50

Magnetite concentrations were calculated using an empirically-derived value for SIRM SP/SD magnetite of 13.8 Am^2^ kg^−1^, for interacting, mixed single domain (SD) and superparamagnetic (SP) magnetite, of mean particle size 31 nm^54^, rather than the ‘conventional’ SIRM magnetite value of 46 Am^2^ kg^−1^, applicable only to pure, non-interacting, single domain (~ 50 nm) magnetite particles. The number of magnetite particles/g dry tissue can then be estimated by dividing the mass of magnetite (per 1 g dry tissue wt) by the mass of 1 magnetite particle (8.07224^–11^ µg). In the (unlikely) case that all of the brain ferrimagnetic material was maghemite, then the maghemite mass concentrations would be 15% higher.

Critically, for the 5 brain regions (cerebellum, entorhinal cortex, frontal, occipital and temporal lobes) from the Manchester cases (n = 30), there is no evidence of homogeneity of magnetite/maghemite distribution; the regional content of brain magnetite/maghemite varies from one individual to another (Fig. 2). Nor is there any evidence for increasing brain ferrimagnetic content or iron content with age for persons aged between 80 and 98 years.

**Figure 3:**
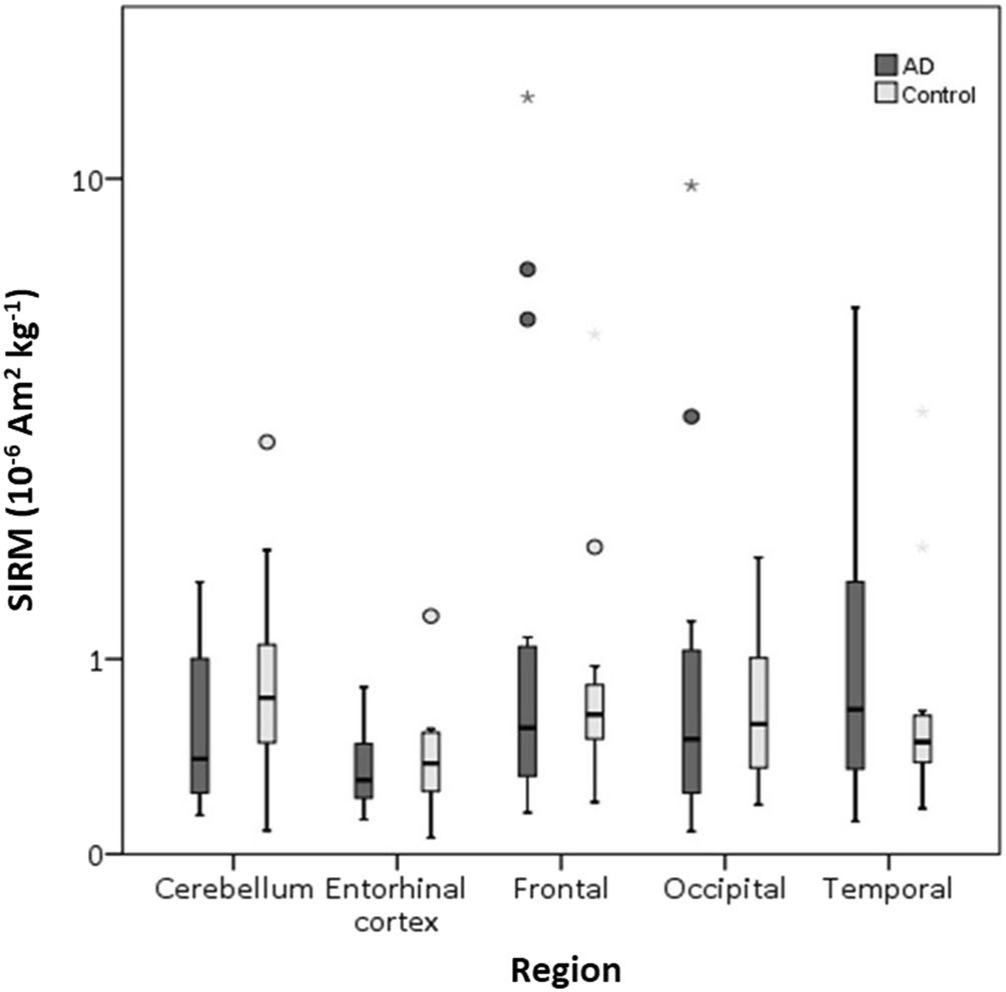
Mass-normalised SIRM values of human brain regions from AD and control brains from Manchester, UK. Box plot represents room temperature (293 K) measurements of freeze-dried human tissue from AD or control brains from the cerebellum (n = 17 AD, 11 Control), entorhinal cortex (n = 17 AD, 9 control), frontal lobe (n = 19 AD, 11 control), occipital lobe (n = 18 AD, 10 control), and temporal lobe (n = 18 AD, 11 control). Outliers (open circle) are more than 1.5× the interquartile range, extremes (*) are more than 3× the interquartile range.

Comparison of AD regions with their control region counterparts showed no significant difference in ferrimagnetic concentration (Independent Mann Whitney–U test) (Fig. 3, Table 1, Supplementary Table S3). Examination of all Manchester cases showed higher concentrations of magnetite/maghemite in the frontal lobe compared to the entorhinal cortex (one-way ANOVA on log-transformed data with Tukey’s post hoc, p = 0.021) (Fig. 3, Supplementary Fig. S2). Higher magnetic concentrations were seen in the frontal lobe of female AD cases compared to female control cases; however, no statistical difference was seen when comparing any region between AD and control cases (as a whole or stratified by sex) (Kruskal–Wallis test on untransformed data) (Supplementary Table S3). Analysis of the data excluding the occipital sample from case 15/29, which has an unusually high NRM, shows higher ferrimagnetic concentrations in both the frontal and temporal lobes compared to the entorhinal cortex (one-way ANOVA on log-transformed data with Tukey’s post hoc, p = 0.015 and p = 0.049 respectively). Given the observed variability in magnetic content, a larger number of AD and control cases (approximately 93 in each group) would be needed for robust statistical analysis.

**Figure 4:**
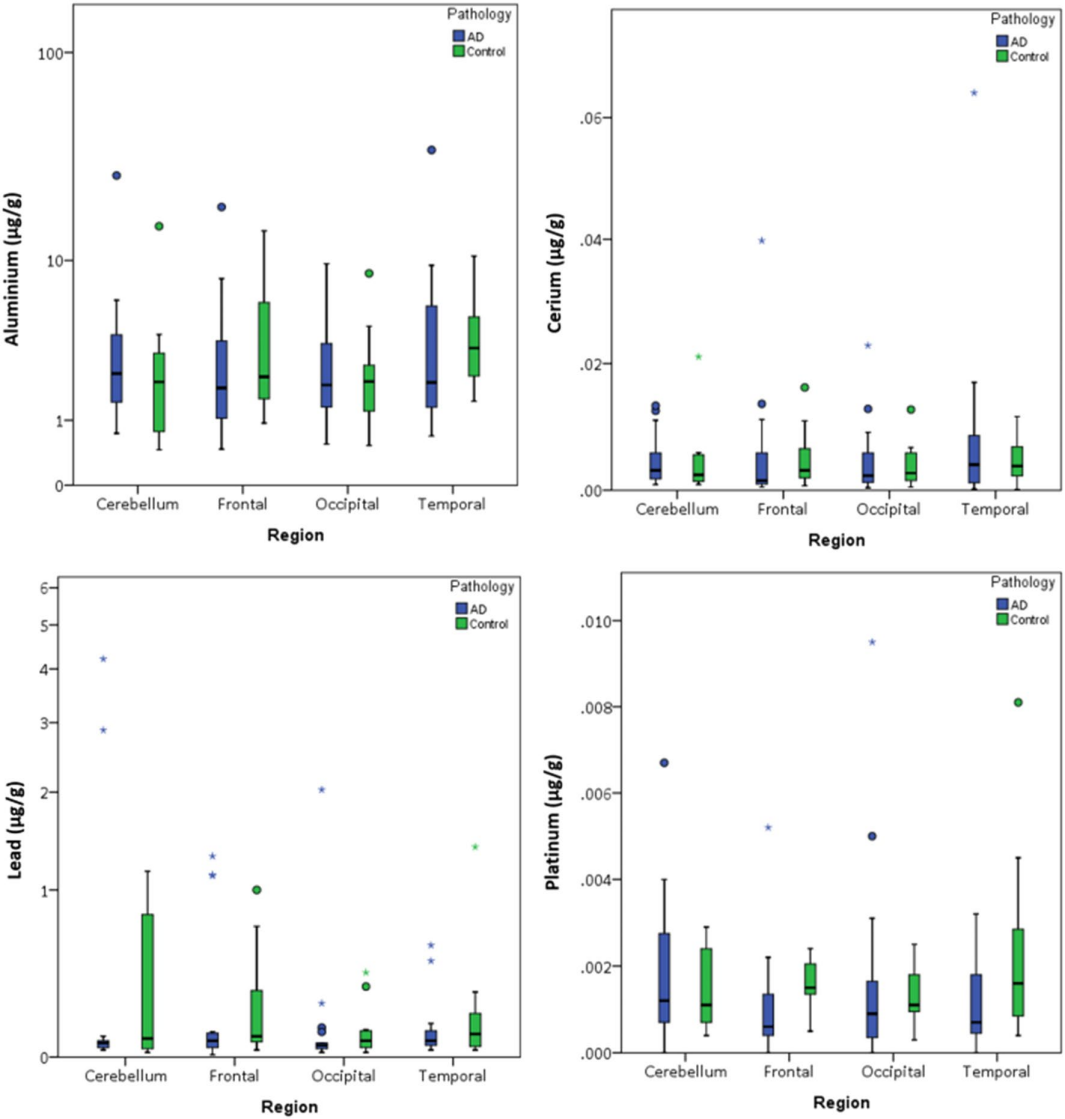
Mass-normalised concentrations of exogenous metals in human brain regions from AD and control brains from Manchester Brain Bank, UK. Box plots represent ICP-MS measurements of aluminium, cerium, lead and platinum in freeze-dried human tissue from AD (n = 19) or control (n = 11) brains from the cerebellum, frontal lobe, occipital lobe, and temporal lobe. Outliers (open circle) are more than 1.5× the interquartile range, extremes (*) are more than 3× the interquartile range.

**Figure 5:**
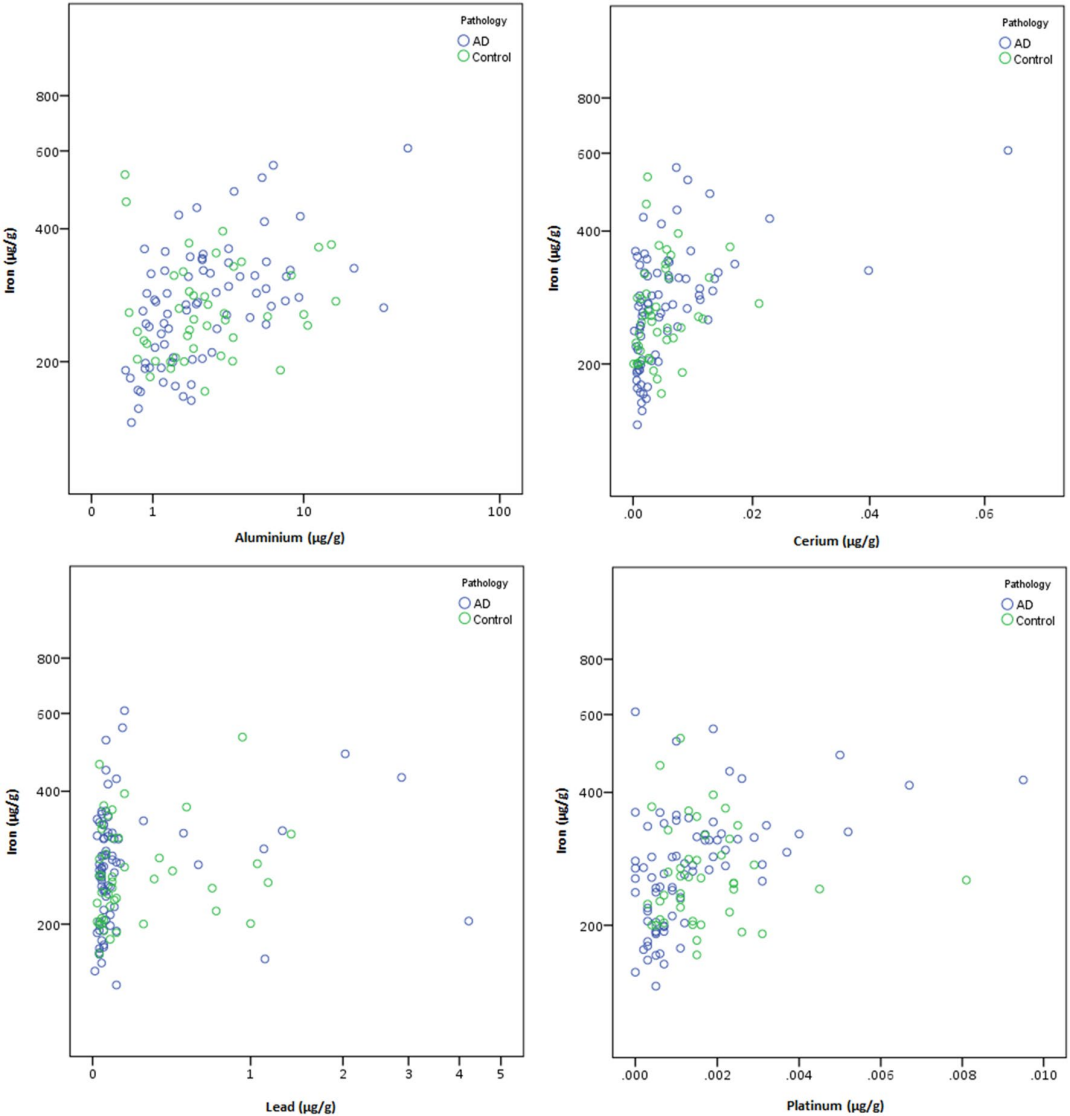
Mass-normalised concentrations of exogenous metals in human brain regions from AD and control brains from Manchester Brain Bank, UK. Scatter plots represent ICP-MS measurements of aluminium, cerium, iron, lead and platinum in freeze-dried human tissue from AD (n = 19) or control (n = 11) brains from the cerebellum, frontal lobe, occipital lobe, and temporal lobe.

As with the magnetite/maghemite concentrations, no significant differences were seen when comparing regional concentrations of exogenous metals in AD cases compared to control cases (Figs. 4, 5, Supplementary Tables S4–S7).

We can usefully compare the concentration of cerebellar magnetite/maghemite in our elderly Manchester cohort with previously reported data for a separate, much younger population, with lifelong residence in Metropolitan Mexico City (MMC)^31^. The MMC samples were freeze-dried and then measured, in Lancaster’s Centre for Environmental Magnetism and Palaeomagnetism, in the same way as the Manchester samples^31^. Mass-normalized SIRMs of MMC cerebellum samples ranged from 0.20 to 36.23 × 10^−6^ Am^2^ kg^−1^ with a median of 2.25 × 10^−6^ Am^2^ kg^−1^, equating to ~14 to 2625 ng of magnetite g^−1^ tissue with a median of 163 ng of magnetite g^−1^ tissue^31^. This is equivalent to a range of 0.18 to 32.52 x 10^9^ particles g^−1^ with a median of 2.02 × 10^9^ particles g^−1^. Comparison of all cases from Mexico City showed significant difference between the 3 regions measured (Kruskal–Wallis test p = 0.0114), specifically between the substantia nigra and cerebellum (Kruskal–Wallis, pairwise comparison with Bonferroni correction p = 0.008). The highest concentration of magnetite/maghemite was found in the cerebellum, followed by the tectum/tegmentum and the substantia nigra.

**Figure 6:**
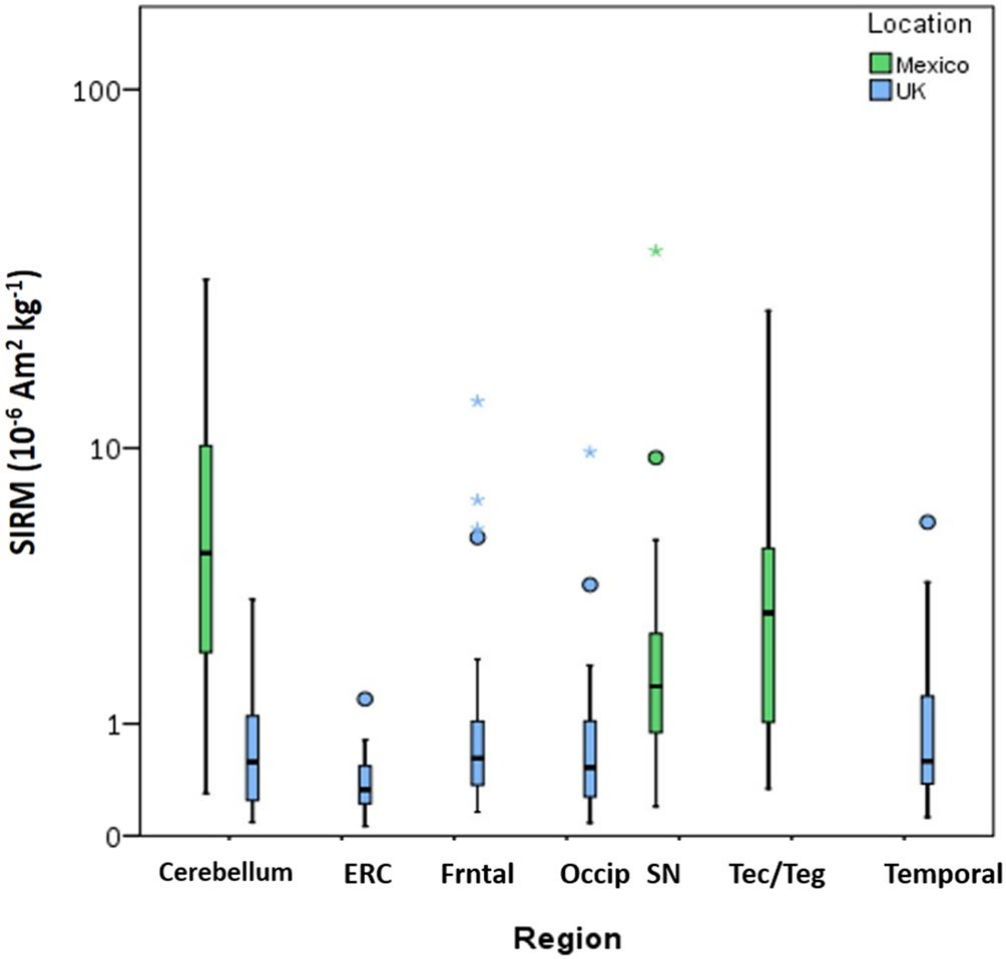
Box plot of mass-normalised SIRM values of human brain regions from Manchester, UK and Mexico City, Mexico. Box plots depict the mass-normalised (freeze-dried weights) saturation isothermal remanence magnetisations (SIRMs) of human brain tissue samples measured at 293 K from the cerebellum, entorhinal cortex (ERC), frontal lobe (Frntal), occipital lobe (Occip), and temporal lobe of cases from Manchester, UK, (blue) along with the cerebellum, substantia nigra (SN), and tectum/tegmentum (Tec/Teg) from Mexico City, Mexico (green)^31^. Outliers (open circle) are more than 1.5× the interquartile range, extremes (*) are more than 3× the interquartile range.

In contrast to the Manchester samples, samples from Mexico City^31^ were from forensic cases (sudden deaths, no brain injury), are a much younger population (aged 12–85 years, mean age 29 years; compared to Manchester: aged 80–98 years, mean age 89 years), and are primarily male. The MMC tissue samples also display neurological damage, previously linked with exposure to high levels of particulate air pollution^55^. Significantly higher magnetite/maghemite concentrations were found in the Mexico City samples compared to the Manchester samples (Independent samples Mann–Whitney U test on untransformed data, p < 0.001). The ferrimagnetic concentrations in the Mexico City samples (all regions) are on average ~5× higher than those of their Manchester Brain Bank counterparts (Fig. 6).

## Discussion

Discrete nanoparticles of the mixed Fe^2+^/Fe^3+^ iron oxide, magnetite, and maghemitised magnetite have been found in senile plaques e.g.^26^ and within the frontal cortex^21^, globus pallidus^56^, temporal lobe^49^, superior temporal gyrus^51^, brainstem^31,47^, cerebellum^31^, and hippocampus^57^ of the human brain, but any relationship between these ferrimagnets and NDD is as yet poorly understood. Using SQUID magnetometry on fresh-frozen human tissue samples, we find a heterogenous distribution of magnetite/maghemite across the brain regions sampled from AD and control cases from northern England. Magnetite/maghemite concentrations are significantly higher in the frontal and temporal lobes (particularly affected in AD) compared to the entorhinal cortex. Conversely, no significant difference either in brain magnetite/maghemite or in exogenous metal (Pb, Pt, Ce, Al) concentrations was found between AD and control cases in this elderly northern England cohort.

Notable is that significantly higher ferrimagnetic concentrations are present in the cerebellum of younger cases from Mexico City^31^ compared to the much older northern England cases investigated here.

Given the variability in regional distribution of magnetic particles, across elderly AD and control groups from northern England (Fig. 2), we find no evidence for genetic control of the magnetite/maghemite distribution in the human brain. In contrast, a recent study of seven whole brains found an identical distribution pattern of magnetic particles across seven studied brains, thence attributed to endogenous, genetically-controlled formation of magnetite/maghemite^47^. Their SIRMs are systematically much lower than those reported here (our MBB samples are on average ~ 9× more magnetic, and the Mexico City cerebellar samples are on average ~ 61× more magnetic^31^) (Supplementary Fig. S3). Gilder et al.^47^ further suggest that the maximum values they observe, for the brainstem and cerebellum—i.e., distal from the olfactory bulb—cannot be accounted for by intake of exogenous ferrimagnetic particles via the nasal route. However, not only are nanoparticles likely to be transported through the brain^58^, but the brainstem may have an additional entry portal via the gut wall and neuroenteric system^31^.

One explanation for Gilder et al.’s low SIRM values is their use of formalin-fixed tissue; 10% formaldehyde was replenished over a storage interval of several decades. Evidence exists for magnetite dissolution by formalin in human tissue; e.g. ~ 50% reduction in SIRM after 1 week in formalin^48^. The storage duration may be important. No changes in ferrimagnetic concentration were reported for paired frozen and fixed tissue samples after 5 months in formalin^50^. Storage for several years in formalin caused transformation from ferritin-like iron to non-ferritin iron as observed by Mössbauer spectroscopy^59^, whereas storage for 1 week did not change the magnetic particle size, as measured by SQUID magnetometry^48^. It seems possible that Gilder et al.’s SIRM values—of the order of 10^–8^ to 10^−7^ Am^2^ kg^−1^—may represent only the remaining fraction of magnetite/maghemite resisting dissolution or transformation during the decades-long period of brain storage in formalin^47^. In contrast, the tissue samples here have been subjected only to freeze-drying; their measured, room-temperature SIRMs (of the order of 10^–5^ to 10^–8^ Am^2^ kg^−1^) are equal to or less than other published SIRM data on human brain tissue (Supplementary Fig. S4)^20,21,50,51,60^.

A previous magnetic study reported higher magnetite concentrations in the temporal lobe of female AD patients compared to controls^51^; similarly, a meta-analysis found higher iron content in the temporal lobe of AD patients compared to controls^9^. Here, higher magnetite/maghemite concentrations were evident in the frontal and temporal lobes. However, we found no significant difference in magnetite/maghemite concentration between the AD and control samples from northern England, U.K. Based on the variability in brain ferrimagnetic content observed here, larger numbers of samples (~ 100 per group) would be required for robust statistical analysis.

For the MBB cases (i.e., AD + controls), higher magnetite/maghemite concentrations were found in the frontal and temporal lobes compared to the entorhinal cortex (Fig. 3, Supplementary Fig. S2), and highest magnetite/maghemite concentrations in the frontal lobes of two female AD cases. Ferrimagnetic content may be elevated in the frontal lobe due to its proximity to the olfactory bulb, a proposed entry route^21,40,61^ of environmental/airborne magnetite^20^. MNP concentrations are also high in the temporal lobe, the cortical region which first shows AD pathology—enriched in both senile plaques and neurofibrillary tangles, the two key pathological markers of AD. Plaques also occur in normal ageing, and MNPs appear to associate with senile plaques^26,29^.

The entorhinal cortex, another brain region to show early AD pathology^53^, receives input from the olfactory bulb, also an early-affected region. Olfactory dysfunction is a possible biomarker for AD development, with correlation to tau biomarkers and significant olfactory dysfunction seen in population studies, e.g.^62^. Pollution NPs have been reported in the olfactory bulb (e.g.^63^), and nasal mucosa, with the olfactory bulb a proposed entry route to the brain^21^. Here, the higher magnetite/maghemite concentrations seen in temporal and frontal lobes, compared to the entorhinal cortex, may reflect “nose to brain” transport pathways. Translocation of 10–550 nm graphene oxide (GO) nanosheets from the olfactory bulb to distal sites (including cerebellum, hippocampus, striatum, midbrain, pons and medulla) has recently been shown in mice, attributed to a combination of transcellular and paracellular movement of particles through and/or between olfactory epithelial cells^58^. GO nanosheets were found in the olfactory bulb 3 h after administration, and in more distal structures (including cortex, hippocampus, cerebellum) within 24 h.

It is notable that, in addition to variable ferrimagnet concentrations, brain samples from both our AD and control cases contain varying concentrations of metal species (Pb, Al, Ce, Pt) of unequivocally exogenous origin. As with the magnetite/maghemite concentrations, there is no significant difference between the exogenous metal (Pb, Al, Ce, Pt) contents of our N. England AD and control brain regions (Figs. 4–5, Supplementary Tables S4–S7). Similarly non-physiological metal species, including vanadium, nickel, manganese, chromium and lead, were reported in frontal lobe samples from Mexico City, with significantly higher manganese, nickel and chromium in Mexico City brains compared to less-polluted controls (from Veracruz, Mexico^64^).

The brain should be protected from incursion of such metals via the tight junctions of the blood–brain barrier (BBB). That both the AD and non-AD cases here contain readily-measurable concentrations of exogenous metals (including unequivocally traffic-derived Pt and Ce) indicates variable levels of exposure to similar pollutants, and, critically, suggests the likely loss of BBB integrity across both of these elderly groups (aged 80 to 98 years at death). The timing of any BBB breakdown in the investigated individuals is unknown but may have occurred at a relatively late life stage. It is established that BBB permeability is greater in older (aged 55–91 years) cognitively normal (CN) individuals compared to young (aged 23–47 years) CN individuals, and even greater in the mildly cognitively impaired (aged 55–85 years) compared to older CN individuals^65^. Interestingly, the metal element, Ce, present in all our Manchester cases, only appeared in the urban particulate pollution ‘mix’ from 1998 onwards (i.e. when our investigated individuals were ~ 60 years or older), when it was introduced as an anti-knock fuel additive^30^. Lead was removed from vehicle fuel in the UK in 1999, but remains a common component of airborne particulate pollution because it is widely used in vehicle ignition systems, fuel tanks, spark plugs etc.^66^. The hypothesized loss of BBB integrity suggested across all of our elderly Manchester cases, i.e. both AD and ‘controls’, would obscure any possible relationships between metal pollution exposures in earlier life and subsequent development of neurological damage.

Ferrimagnetic concentrations are significantly lower (~ 9× lower) in the cerebellar samples from the elderly MBB cases compared with much younger Mexico City cases^31^. Given the possibility of particle dissolution and clearance by the brain^30^, the location/environment to which a young person is chronically exposed may be reflected—as a ‘snapshot’, at post mortem—by the composition and concentration of exogenous metals present^20^, such as platinum, cerium, and co-associated MNPs. Mexico City is highly polluted (PM_2.5_ 32 µg/m^3^ on average for 2009) compared to e.g. Manchester, U.K. (PM_2.5_ 12 µg/m^3^ on average for 2009). Studies have shown co-association between the abundant, intra-cellular presence of metal-rich nanoparticles and neurodegeneration, including brain inflammation and markers of AD pathology, even in children as young as 11 months e.g.^31,55^. As noted above, BBB integrity is known to deteriorate with age and NDD. Yet the Mexico City cases, which comprise a much younger cohort (mean age 29 years) than the Manchester cases (mean age 89 years), have significantly higher concentrations of brain magnetite/maghemite. In the absence of environmental insult, the BBB integrity of young cases should be relatively intact, or minimally altered^67^. The high concentrations of magnetite/maghemite and metals seen in the young Mexico City brains thus most likely reflect their high lifelong and/or recent exposure to air pollution via inhalation and/or ingestion^31^. Age-matched controls from a less-polluted city (Veracruz, Mexico) show no such neuropathology and much lower brain nanoparticle content.

Levels of exposure to airborne, iron-rich and co-associated metal-rich pollution nanoparticles will be spatially and temporally variable not only across different urban areas but also between different cities worldwide, dependent on emission sources (traffic, domestic and industrial), and population proximity to those sources. Annual mean concentrations of airborne magnetite nanoparticles in Beijing, for example, have recently been reported as 75.5 ± 33.2 ng m^−3^ (~ 0.1% of PM_2.5_ mass), with strong seasonal variations^68^. Magnetite concentrations up to 0.8% of PM_2.5_ mass have been reported in Thessaloniki, the second largest city in Greece^69^. Given such reported variations, both epidemiological and forensic analyses would be valuable in identifying any specific dose–response associations between exposures to airborne magnetic nanoparticles and both neurodegenerative and cardiovascular deficits^30,33^.

Given the probability of BBB degradation in the very elderly, and the possibility of at least some dissolution and clearance from the brain, it is unlikely that exogenous pollution particles merely accumulate in the brain with time/age. Hence, there may be little correlation in the very elderly between brain magnetite/maghemite and metal content either with age or AD status. Conversely, investigation of young, variably-exposed cases, with relatively intact BBB, may provide the information key for understanding the sub-cellular impacts of air pollution particles on the brain in early life, and the neurological damage manifested often decades later in the life course.

## Conclusions

1. We find a heterogenous distribution of magnetite/maghemite across AD and cognitively normal brains from northern England, as determined by SQUID magnetometry. The observed heterogeneity negates a previous suggestion of universal, genetic control on the distribution of magnetite/maghemite within the brain. Conversely, such variations are consistent with different levels of exposure to and/or clearance of exogenous magnetic nanoparticles, which occur in abundance at the roadside, for example, but are also emitted by industry, residential fires, some occupational sources etc.
2. In the northern England cases, highest ferrimagnetic concentrations were found in the frontal lobe in both AD and control cases.
3. There were no significant differences in either the ferrimagnetic content or the concentrations of other metal pollutants (Al, Pb and unequivocally vehicle-derived metals, e.g., Pt and Ce) between the AD and the control cases from northern England. Incursion of such exogenous metals suggests loss of BBB integrity in both elderly groups, with potentially enhanced particulate incursion from a late life stage (i.e. >  ~ 60 years).
4. Compared to the elderly northern England cases, magnetite/maghemite concentrations were significantly higher in the much younger Mexico City cases, where BBB integrity would be expected. Common to the young Mexico City cohort is their lifelong exposure to much higher levels of particulate air pollution compared to their elderly northern England counterparts.
5. Investigation of any causal role of magnetite/maghemite and other metal-bearing air pollution nanoparticles should focus on young, apparently healthy, variably-exposed cohorts, in order to identify any associations between early neuropathology and air pollution exposures, especially regarding particulate composition, size distribution, portals of entry (e.g. inhalation vs ingestion), and specific, sub-cellular biological targets. Such information is essential in order to understand the initiation and early development of neuropathology in the decades prior to clinical manifestation of NDD in later life.

## Supplementary Information

Supplementary Information.
