# Supplementary material for: Variation in the concentration and regional distribution of magnetic nanoparticles in human brains, with and without Alzheimer’s disease, from the UK
Source: Sci Rep. 2021 Apr 30;11:9363. doi: 10.1038/s41598-021-88725-3 (PMC8087805; doi:10.1038/s41598-021-88725-3)
Supplement: Supplementary file 1 — Supplementary Information. [file 41598_2021_88725_MOESM1_ESM.pdf]

Variation in the concentration and regional distribution of magnetic nanoparticles in human brains, with and without Alzheimer’s disease, from the UK

Jessica Hammond[1,*], Barbara A. Maher[2,*], Imad A. M. Ahmed[3], and David Allsop[1]

[1]Division of Biomedical and Life Sciences, Faculty of Health and Medicine, Lancaster University, LA1 4YQ, U.K
[2]Centre for Environmental Magnetism and Palaeomagnetism, Lancaster Environment Centre, Lancaster University, LA1 4YQ, U.K.
[3]Department of Earth Sciences, University of Oxford, OX1 3AN, U.K.

[*]jessica.hammond@lancaster.ac.uk, b.maher@lancaster.ac.uk

Supplementary Information

Supplementary methods

The measurement of (relatively) weakly magnetic samples of a biological nature presents several experimental challenges. At every stage, we have reduced and quantified any potential contamination (Supplementary Table S1). First, an appropriate, magnetically-clean environment was selected for sample preparation; a biological safety cabinet in a class III laboratory. Cabinet air throughflow was sampled using a Leland Legacy pump (SKC, Dorset UK) at 7.5L/min through a magnetically-'clean' 1 μm PTFE filter, in order to quantify any magnetic 'background' associated with that environment.

Supplementary Table S1 Sampling Air Quality

Name	Location	SIRM (x 10^{-10} Am^2)	SIRM (x 10^{-10} Am^2/m^3)	SIRM (x10^{-10} Am^2/min)
CL3(11/10/18)	Hood	18.43	6.15	0.0460
CL3 (10/05/18)	Hood	9.7	5.44	0.0400
CEMP	Bench	3.36	1.14	0.0086
CL3 (03/10/19)	Hood	1.45	0.57	0.0043

CL3 = biological safety cabinet in class III laboratory, CEMP = Centre for Environmental Magnetism and Palaeomagnetism. The SIRM of clean 1 μm PTFE filters was measured, these were then placed inside the pump and air was sampled for between 3 and 7 hours, which equates to 1.35-3.15m^3 air. The SIRM of the exposed filters was then measured. The values above reflect the change in filter SIRM, post-exposure, at each location, normalised for the volume of air pumped, or per minute of exposure, on the two tissue sampling days. The last two rows represent a random sampling of the sample preparation area (hood) or sample measurement area (CEMP, bench).

The class III laboratory has double filtered air (high efficiency particulate air, HEPA, filters) inside the biological safety cabinet. Tissue samples were exposed within the hood for ~ 3 minutes or less; this translates to potential magnetic accumulation of a minimum 1.20 E-12 Am^2 (i.e., ~ 3-4 times lower than a typical 2G sample holder value) or a maximum 1.38 E-11 Am^2 (~ 3-4 higher than a typical holder value).

In order to minimize contamination of the sample and clingfilm, all handling of clingfilm and samples was carried out in the biological safety cabinet. Clingfilm is necessary in order to secure and protect the freeze-dried samples which are fragile and will crumble if repeatedly manipulated. For each tissue sample measurement, the specific piece of clingfilm was pre-measured and demagnetised (pieces with an SIRM $\geq 2 \times 10^{-10}$ Am^2 were rejected and not used for measurement). Clingfilm was cut from the centre of the roll using a ceramic knife. The SIRM of the clingfilm, ave. ~1 x 10^{-10} Am^2, is subtracted in order to isolate the tissue SIRM. On average, the clingfilm contributes ~23% of the total measured SIRM (i.e. sample + pot + clingfilm). Tissue samples were measured in non-magnetised pots; the NRM of an empty pot (~6 x 10^{-12} Am^2) is weaker than a typical 2G sample holder value.

Cutting surfaces and ceramic knives were cleaned with 70% ethanol prior to use and between samples. To quantify any possible contamination from the ethanol, SIRM measurements were made on unfiltered and 1, 2 or 3x filtered 70% ethanol, as well as unfiltered dH_2O. SIRMs were measured at 77K on a JR6A magnetometer (noise level 5 x 10^{-11} Am^2); the SIRM of each solution, approximately 2g (~2-2.3ml), was unmeasurable.

The outer surfaces of each sample were trimmed to remove any potential metal contaminants from the autopsy process, such as fragments from stainless steel instruments or debris from metal bone saws as per [1]. Some large samples and those with irregular geometry were further sub-divided into two samples, each sample measured separately and the final SIRM calculated by adding the two values as if it were one whole sample. For a selection of samples, the trimmings were measured as well as the sample. In some cases, trimming made little difference; in other cases, a failure to trim the sample would have made a large contribution to the measured signal, emphasising the need to control for contamination at every stage. Wet/dry ratios (i.e., the mean of the freeze-dried weight divided by the wet weight) ranged from 0.16 for entorhinal cortex samples and 0.19 for all other MBB samples.

Data presented here classes each Manchester case as either 'AD' or 'control'. However, the pathological diagnosis in some cases is not as clear; for example, "probable AD" and "possible AD". Data were also categorised as control, AD or "intermediate" if the case did not clearly belong to either the control or AD group and analysed using this classification system. There was no significant difference in ferrimagnetic concentration between AD and controls when using a 3 group classification (AD, Control, intermediate), a 2 group classification (all cases designated as either control or AD) or a 2 group

classification system omitting the intermediate cases (definite AD and definite controls only).

We note that samples from Manchester originated from the right hemisphere, whilst the previously-published Mexico City samples [4] originated from the left hemisphere. Limited tissue availability prevented us from using the same hemisphere from both populations. It is possible that variation exists between the left and right hemispheres in terms of magnetite/maghemite concentration and pathology. However, MRI studies have shown no interhemispheric differences in iron concentrations [2], and pathological examination of both AD and control hemispheres (as a whole group) showed similar Braak NFT staging and Aβ deposition but increased vascular lesions in the left hemisphere [3]. In contrast, Gilder et al. report higher ferrimagnetic concentrations in the left hemisphere of the cerebral cortex than the right hemisphere in six of their seven cases [1].

Supplementary Table S2 Summary case information Manchester, UK.

Case	Sex	Age	Pathological Diagnosis	State
09/15	F	89	AD	AD
09/22	F	90	Possible AD	AD
09/37	M	90	Age changes only	Cont.
10/08	F	87	AD	AD
10/13	F	85	AD	AD
10/16	M	91	Moderate SVD	Cont.
10/26	M	84	Moderate SVD	Cont.
10/40	M	86	AD	AD
12/09	F	87	Mild AD pathology in temporal lobe	AD
12/23	M	95	Age changes only	Cont.
12/33	F	81	Probable AD	AD
12/34	F	80	Probable AD	AD
13/09	F	85	AD	AD
13/10	F	85	AD	AD
14/01	F	93	Probable AD	AD
14/04	F	87	Age changes only	Cont.
14/07	F	95	Probable AD	AD
14/11	M	91	Mild SVD	Cont.
14/20	F	90	Age changes only	Cont.
14/46	F	94	Age changes only	Cont.
15/01	M	90	Age changes only	Cont.
15/19	F	98	Possible AD	AD
15/29	M	81	AD	AD
15/31	M	91	Age changes only	Cont.
15/42	M	89	Incipient AD	Cont.
15/48	F	81	AD	AD
16/01	M	90	Possible AD	AD
16/08	M	88	Incipient AD	AD
16/13	M	91	AD	AD
16/14	F	92	AD	AD

A selection of 30 brains from elderly individuals were obtained from the Manchester Brain Bank. The pathological diagnosis of these cases can be summarised as; 10 Alzheimer’s Disease (AD), 7 controls (cont.) (age changes only), 4 probable AD, 3 possible AD, 2 incipient AD, 2 moderate small vessel disease (SVD), 1 mild SVD and 1 mild AD pathology in the temporal lobe.

Supplementary Table S3 Regional distribution of magnetic remanence carriers in Manchester Brain Bank (MBB) Alzheimer's disease and control cases. Freeze dried (originally fresh frozen) brain tissue samples from the cerebellum, entorhinal cortex (EC), frontal, occipital and temporal lobes were exposed to a saturating DC field of 1 T and their saturation isothermal remanence magnetisation (SIRM) measured. Cases were classified as either control (cont.) or Alzheimer's disease (AD) based on their pathological diagnosis.

**Region**	**Samples (n)**	**SIRM range ($10^{-6}Am^2 kg^{-1}$)**	**Mean SIRM ($10^{-6}Am^2 kg^{-1}$)**	**Median SIRM ($10^{-6}Am^2 kg^{-1}$)**	**Mean magnetite (μg/g)**	**Mean (10^9) particles per g tissue**
Cerebellum AD	17	0.15-1.63	0.63	0.40	0.05	0.57
Cerebellum Cont.	11	0.09-3.31	1.01	0.74	0.07	0.91
EC AD	17	0.13-0.81	0.38	0.30	0.03	0.34
EC Cont.	9	0.06-1.33	0.45	0.38	0.03	0.40
Frontal AD	19	0.16-13.69	1.86	0.57	0.13	1.67
Frontal Cont.	11	0.20-5.33	1.13	0.64	0.08	1.01
Occipital AD	18	0.08-9.73	1.22	0.51	0.09	1.10
Occipital Cont.	10	0.19-1.87	0.73	0.59	0.05	0.66
Temporal AD	18	0.12-5.96	1.14	0.67	0.08	1.02
Temporal Cont.	11	0.17-3.80	0.89	0.49	0.06	0.80

Supplementary Table S4 Metal concentrations in the cerebellum of MBB Alzheimer's disease and control cases.

Pathology	Case	Region	Aluminium	Cerium	Iron	Lead	Platinum
AD	09/15	Cerebellum	2.54	0.0019	321.95	0.09	0.0040
AD	09/22	Cerebellum	5.00	0.0125	251.83	0.04	0.0031
Control	09/37	Cerebellum	1.54	0.0059	313.48	0.09	2.2009
AD	10/08	Cerebellum	2.50	0.0031	203.23	4.21	0.0005
AD	10/13	Cerebellum	1.20	0.0013	231.30	0.06	0.0011
Control	10/16	Cerebellum	2.01	0.0043	371.22	0.05	0.0004
Control	10/26	Cerebellum	3.54	0.0014	248.54	1.16	0.0024
AD	10/40	Cerebellum	0.84	0.0009	198.48	0.08	0.0007
AD	12/09	Cerebellum	2.13	0.0042	202.37	0.03	0.0012
Control	12/23	Cerebellum	0.48	0.0022	460.43	0.03	0.0006
AD	12/33	Cerebellum	0.79	0.0046	260.53	0.04	0.0012
AD	12/34	Cerebellum	3.13	0.0057	237.42	0.06	0.0000
AD	13/09	Cerebellum	6.06	0.0047	415.17	0.07	0.0067
AD	13/10	Cerebellum	2.29	0.0073	446.60	0.06	0.0023
AD	14/01	Cerebellum	2.27	0.0009	270.21	0.05	0.0002
Control	14/04	Cerebellum	0.68	0.0013	202.61	0.02	0.0007
AD	14/07	Cerebellum	5.45	0.0110	285.95	0.05	0.0019
Control	14/11	Cerebellum	2.69	0.0057	241.34	0.69	0.0024
Control	14/20	Cerebellum	0.46	0.0024	530.43	0.93	0.0011
Control	14/46	Cerebellum	14.83	0.0211	274.13	1.06	0.0029
Control	15/01	Cerebellum	2.59	0.0014	281.04	0.03	0.0015
AD	15/19	Cerebellum	0.74	0.0017	170.89	0.03	0.0005
AD	15/29	Cerebellum	26.20	0.0025	265.09	0.03	0.0014
Control	15/31	Cerebellum	0.87	0.0009	219.66	0.08	0.0011
Control	15/42	Cerebellum	3.99	0.0054	328.71	0.04	0.0008
AD	15/48	Cerebellum	2.09	0.0024	177.40	0.05	0.0011
AD	16/01	Cerebellum	0.96	0.0024	316.42	0.07	0.0029
AD	16/08	Cerebellum	2.50	0.0060	340.20	0.03	0.0007
AD	16/13	Cerebellum	1.68	0.0017	429.83	2.88	0.0026
AD	16/14	Cerebellum	6.19	0.0133	292.79	0.06	0.0037

ICP-MS was conducted on fresh/frozen brain tissue samples from the cerebellum to determine concentrations (µg/g dry tissue) of aluminium, cerium, iron, lead, and platinum. Cases were classified as either control or Alzheimer's disease (AD) based on their pathological diagnosis. *Excluded from analysis as an extreme outlier.

Supplementary Table S5 Metal concentrations in the frontal lobe of MBB Alzheimer's disease and control cases.

Pathology	Case	Region	Aluminium	Cerium	Iron	Lead	Platinum
AD	09/15	Frontal	2.53	0.0007	350.36	0.07	0.0013
AD	09/22	Frontal	0.70	0.0015	156.49	0.01	0.0000
Control	09/37	Frontal	0.94	0.0040	184.82	0.08	0.0015
AD	10/08	Frontal	3.71	0.0111	296.24	1.12	0.0022
AD	10/13	Frontal	2.09	0.0014	163.26	0.04	0.0007
Control	10/16	Frontal	10.02	0.0109	255.93	0.09	0.0016
Control	10/26	Frontal	1.06	0.0007	200.60	1.00	0.0014
AD	10/40	Frontal	0.57	0.0007	145.48	0.11	0.0005
AD	12/09	Frontal	6.63	0.0090	267.12	0.04	0.0018
Control	12/23	Frontal	1.59	0.0027	204.31	0.05	0.0014
AD	12/33	Frontal	1.27	0.0012	244.31	0.07	0.0006
AD	12/34	Frontal	18.44	0.0398	325.64	1.30	0.0052
AD	13/09	Frontal	2.90	0.0037	209.90	0.08	0.0009
AD	13/10	Frontal	1.82	0.0022	166.75	1.13	0.0003
AD	14/01	Frontal	1.05	0.0008	215.35	0.05	0.0003
Control	14/04	Frontal	2.18	0.0031	249.52	0.09	0.0024
AD	14/07	Frontal	8.05	0.0136	311.85	0.10	0.0020
Control	14/11	Frontal	12.04	0.0056	363.31	0.09	0.0013
Control	14/20	Frontal	3.41	0.0075	395.07	0.15	0.0019
Control	14/46	Frontal	14.06	0.0162	368.40	0.51	0.0022
Control	15/01	Frontal	1.44	0.0008	199.99	0.03	0.0005
AD	15/19	Frontal	1.28	0.0005	218.92	0.10	0.0003
AD	15/29	Frontal	0.47	0.0006	191.17	0.02	0.0005
Control	15/31	Frontal	2.17	0.0012	214.26	0.72	0.0023
Control	15/42	Frontal	3.48	0.0030	257.34	0.04	0.0013
AD	15/48	Frontal	0.93	0.0011	193.87	0.05	0.0005
AD	16/01	Frontal	1.04	0.0012	276.23	0.11	0.0012
AD	16/08	Frontal	3.61	0.0043	255.26	0.04	0.0000
AD	16/13	Frontal	1.07	0.0025	273.38	0.03	0.0014
AD	16/14	Frontal	6.19	0.0074	242.91	0.08	0.0005

Supplementary Table S5. ICP-MS was conducted on fresh frozen brain tissue samples from the frontal lobe to determine concentrations (µg/g dry tissue) of aluminium, cerium, iron, lead, and platinum. Cases were classified as either control or Alzheimer's disease (AD) based on their pathological diagnosis.

Supplementary Table S6 Metal concentrations in the occipital lobe of MBB Alzheimer's disease and control cases

Pathology	Case	Region	Aluminium	Cerium	Iron	Lead	Platinum
AD	09/15	Occipital	7.95	0.0025	274.71	0.13	0.0031
AD	09/22	Occipital	2.83	0.0059	317.16	0.02	0.0015
Control	09/37	Occipital	1.96	0.0067	229.05	0.11	0.0011
AD	10/08	Occipital	4.01	0.0128	485.98	2.03	0.0050
AD	10/13	Occipital	1.50	0.0013	199.43	0.03	0.0007
Control	10/16	Occipital	2.02	0.0022	288.43	0.06	0.0021
Control	10/26	Occipital	0.68	0.0010	234.02	0.07	0.0007
AD	10/40	Occipital	0.55	0.0006	183.60	0.04	0.0003
AD	12/09	Occipital	2.05	0.0023	345.71	0.02	0.0010
Control	12/23	Occipital	0.53	0.0020	258.43	0.03	0.0011
AD	12/33	Occipital	0.92	0.0011	239.99	0.05	0.0009
AD	12/34	Occipital	9.60	0.0229	426.80	0.11	0.0095
AD	13/09	Occipital	5.88	0.0091	522.22	0.06	0.0010
AD	13/10	Occipital	2.49	0.0058	342.91	0.25	0.0019
AD	14/01	Occipital	1.58	0.0007	176.10	0.03	0.0002
Control	14/04	Occipital	2.03	0.0040	236.08	0.04	0.0011
AD	14/07	Occipital	4.36	0.0088	311.80	0.05	0.0018
Control	14/11	Occipital	4.45	0.0054	336.82	0.04	0.0025
Control	14/20	Occipital	3.09	0.0063	352.72	0.07	0.0015
Control	14/46	Occipital	8.58	0.0127	313.94	0.12	0.0023
Control	15/01	Occipital	0.81	0.0005	223.32	0.02	0.0003
AD	15/19	Occipital	1.25	0.0013	179.53	0.05	0.0003
AD	15/29	Occipital	1.36	0.0015	256.63	0.03	0.0004
Control	15/31	Occipital	2.17	0.0007	282.42	0.34	0.0013
Control	15/42	Occipital	1.69	0.0027	263.99	0.42	0.0008
AD	15/48	Occipital	0.85	0.0012	243.73	0.04	0.0009
AD	16/01	Occipital	1.52	0.0015	204.38	0.06	0.0003
AD	16/08	Occipital	1.91	0.0054	269.33	0.04	0.0000
AD	16/13	Occipital	0.82	0.0004	360.18	0.04	0.0006
AD	16/14	Occipital	3.09	0.0042	287.09	0.06	0.0010

ICP-MS was conducted on fresh frozen brain tissue samples from the occipital lobe to determine concentrations (µg/g dry tissue) of aluminium, cerium, iron, lead, and platinum. Cases were classified as either control or Alzheimer's disease (AD) based on their pathological diagnosis.

Supplementary Table S7 Metal concentrations in the temporal lobe of MBB Alzheimer's disease and control cases.

Pathology	Case	Region	Aluminium	Cerium	Iron	Lead	Platinum
AD	09/15	Temporal	1.39	0.0002	237.63	0.05	0.0005
AD	09/22	Temporal	6.22	0.0170	336.78	0.05	0.0032
Control	09/37	Temporal	7.46	0.0082	191.31	0.11	0.0031
AD	10/08	Temporal	2.32	0.0066	272.73	0.59	0.0022
AD	10/13	Temporal	1.20	0.0010	193.67	0.03	0.0007
Control	10/16	Temporal	3.96	0.0056	226.83	0.10	0.0006
Control	10/26	Temporal	1.85	0.0001	200.12	0.25	0.0004
AD	10/40	Temporal	0.83	0.0007	192.89	0.11	0.0005
AD	12/09	Temporal	1.35	0.0031	285.63	0.04	0.0004
Control	12/23	Temporal	1.45	0.0034	193.00	0.05	0.0026
AD	12/33	Temporal	1.93	0.0016	261.65	0.10	0.0007
AD	12/34	Temporal	34.70	0.0641	608.76	0.15	0.0000
AD	13/09	Temporal	5.34	0.0076	313.49	0.05	0.0025
AD	13/10	Temporal	1.99	0.0060	311.67	0.12	0.0017
AD	14/01	Temporal	3.71	0.0011	335.14	0.05	0.0003
Control	14/04	Temporal	2.73	0.0038	269.49	0.15	0.0011
AD	14/07	Temporal	8.46	0.0142	322.48	0.07	0.0021
Control	14/11	Temporal	3.32	0.0026	206.06	0.04	0.0006
Control	14/20	Temporal	6.32	0.0116	253.18	0.31	0.0081
Control	14/46	Temporal	10.51	0.0080	241.66	0.09	0.0045
Control	15/01	Temporal	1.82	0.0019	319.96	1.39	0.0017
AD	15/19	Temporal	0.69	0.0012	172.37	0.03	0.0006
AD	15/29	Temporal	1.30	0.0019	355.32	0.04	0.0010
Control	15/31	Temporal	3.93	0.0007	200.53	0.04	0.0016
Control	15/42	Temporal	2.60	0.0047	171.32	0.03	0.0015
AD	15/48	Temporal	6.81	0.0072	556.94	0.14	0.0019
AD	16/01	Temporal	0.87	0.0011	285.57	0.09	0.0009
AD	16/08	Temporal	9.43	0.0112	279.78	0.09	0.0000
AD	16/13	Temporal	1.29	0.0040	321.47	0.49	0.0017
AD	16/14	Temporal	3.71	0.0096	360.64	0.06	0.0000

ICP-MS was conducted on fresh frozen brain tissue samples from the temporal lobe to determine concentrations (µg/g dry tissue) of aluminium, cerium, iron, lead, and platinum. Cases were classified as either control or Alzheimer's disease (AD) based on their pathological diagnosis.

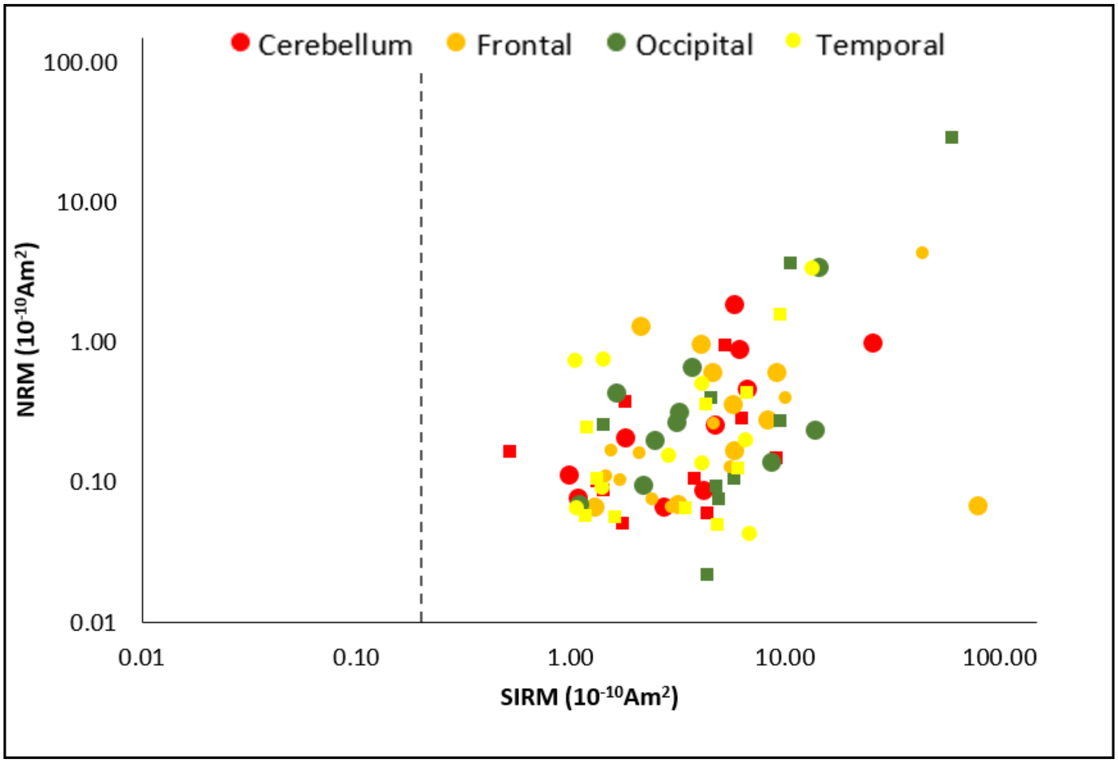

Supplementary Figure S1. NRM/SIRM values of human brain tissue samples**.** The NRM of 83 freeze-dried human brain tissue samples from MBB at 293K ±0.5K. Dashed line indicates the measurement limit (2 x 10^{-11} Am^2) of the 2G SQUID magnetometer (Centre for Environmental Magnetism and Palaeomagnetism, Lancaster University). Circles indicate females, squares indicate males.

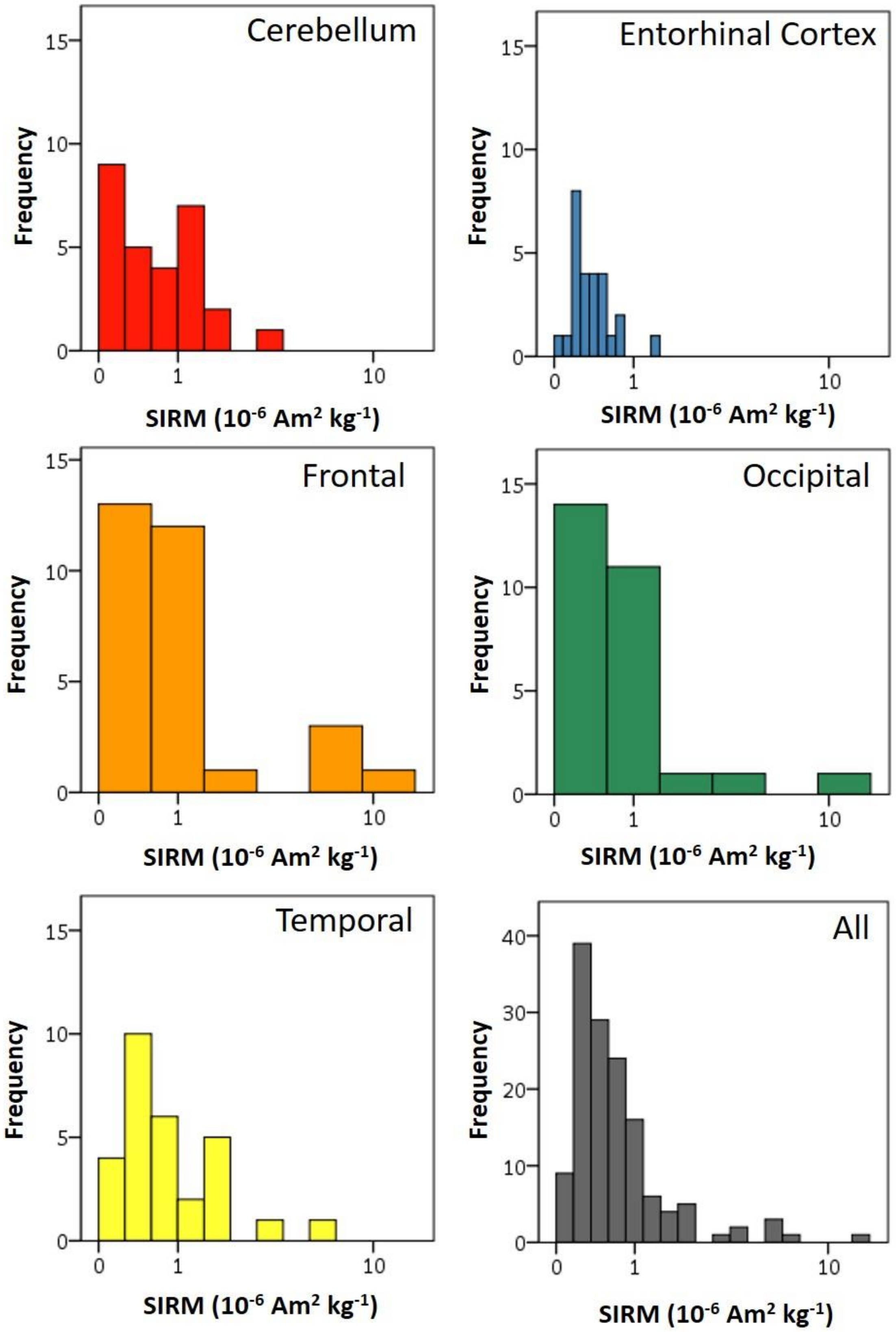

Supplementary Figure S2. Mass normalised saturation isothermal remanence magnetisations of human brain regions from MBB cases, UK. Histograms depict the mass-normalised (freeze-dried weights) SIRM values of human brain tissue samples (Manchester cases) from the cerebellum, entorhinal cortex, frontal lobe, occipital lobe, temporal lobe and all regions.

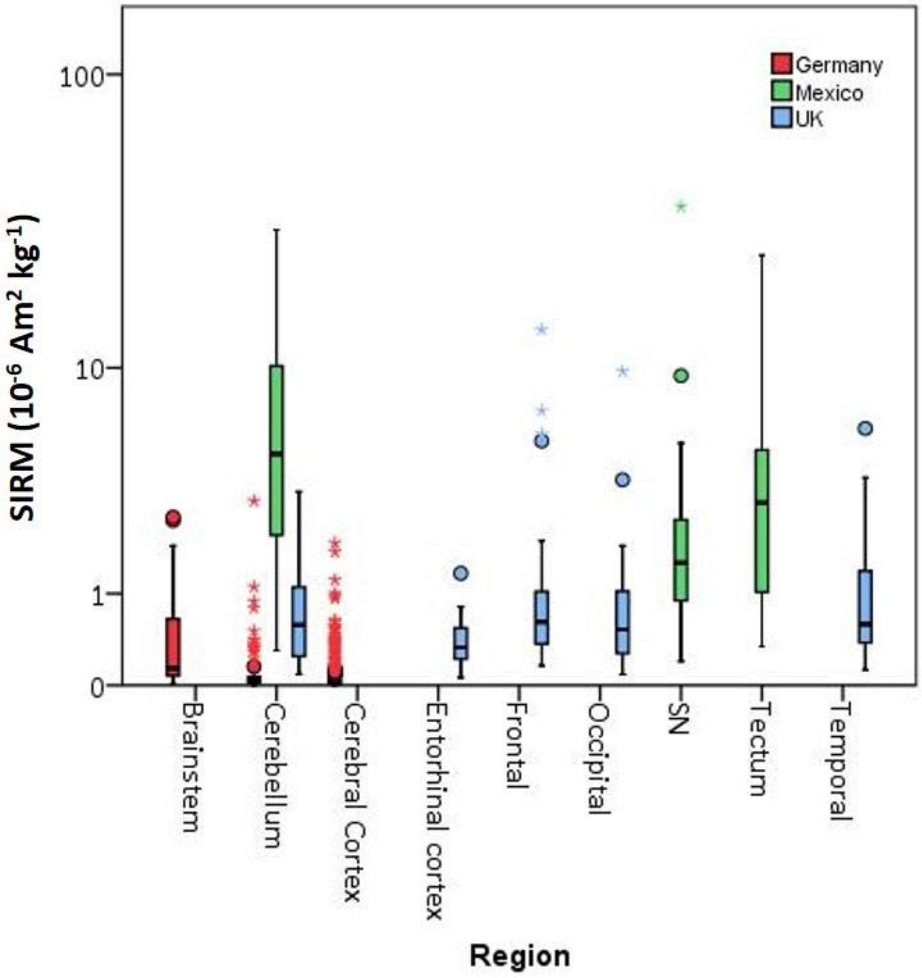

Supplementary Figure S3. Box plot of mass-normalised SIRM values of human brain regions**.** Box plots depict the mass-normalised (freeze-dried weights for Mexico and UK, wet/formalin-soaked weights for Germany) saturation isothermal remanence magnetisations (SIRMs) of human brain tissue samples from the brainstem, cerebellum and cerebral cortex from Germany (red)[1], the cerebellum, substantia nigra (SN) and tectum/tegmentum from Mexico City, Mexico[4], and the cerebellum, entorhinal cortex (EC), frontal lobe, occipital lobe and temporal lobe of cases from Manchester, UK (this study, blue). Outliers (○) are more than 1.5x the interquartile range, extremes (*) are more than 3x the interquartile range.

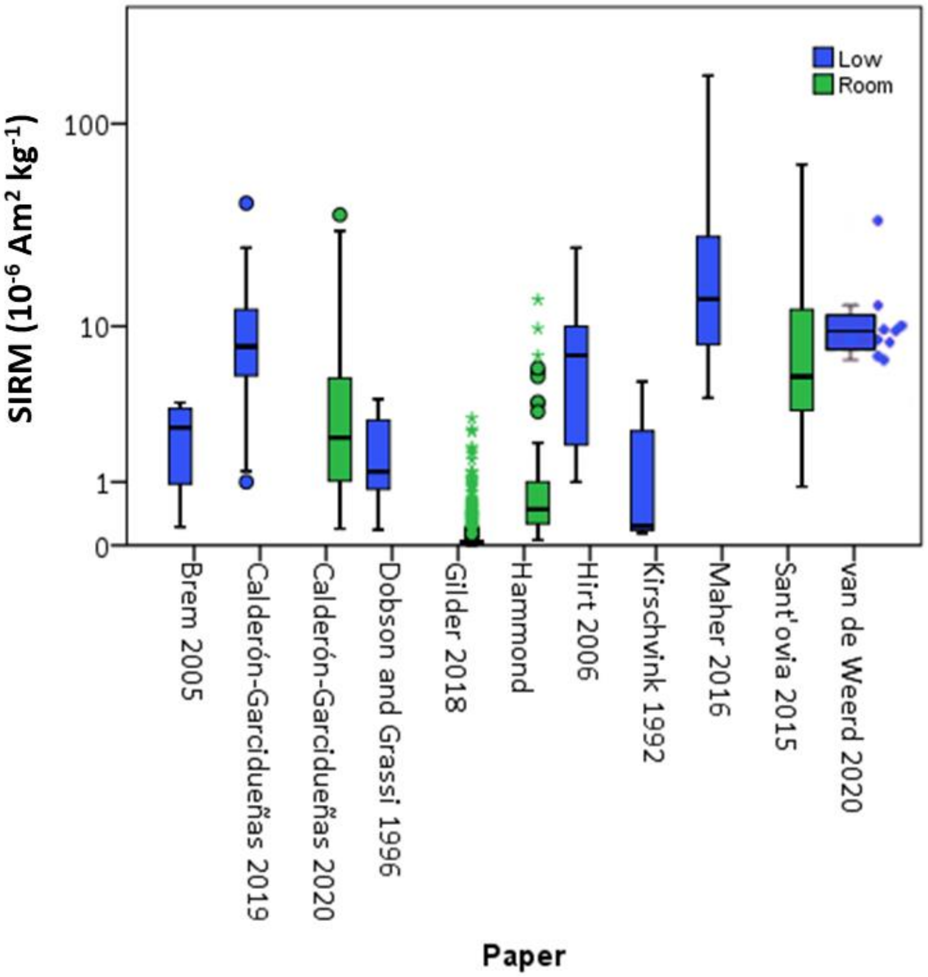

Supplementary Figure S4. Box plot of published mass-normalised SIRM values of human brain regions. Box plots depict the mass-normalised saturation isothermal remanence magnetisations (SIRMs) of human brain tissue samples measured at low (77 K) or room (293 K) temperature. The 'van de Weerd 2020' data were measured at 100K, and represents fresh frozen AD tissue (boxplot taken from [5]). The 'Calderón-Garcidueñas 2019' boxplot represents data from human frontal tissue samples. Data published in [1, 4-12].Outliers (○) are more than 1.5x the interquartile range, extremes (*) are more than 3x the interquartile range. Samples obtained from the following locations: Brem et al., 2005, Zurich, Switzerland; Calderón-Garcidueñas et al., Mexico City and Veracruz, Mexico; Dobson & Grassi, 1996, Zurich, Switzerland; Gilder et al., Heideleberg, Wiesloch, Bayreuth, Germany; Hammond et al., N. England; Hirt et al., Zurich, Switzerland; Maher et al., N England and Mexico City; Sant'ovia et al., Porto, Portugal; Van de Weerd et al., Amsterdam, Netherlands.

Supplementary references

1. Gilder, S.A., et al., *Distribution of magnetic remanence carriers in the human brain.* Scientific Reports, 2018. **8**(1): p. 11363.
2. Acosta-Cabronero, J., et al., *In vivo MRI Mapping of Brain Iron Deposition across the Adult Lifespan.* The Journal of Neuroscience, 2016. **36**(2): p. 364.
3. Giannakopoulos, P., et al., *Interhemispheric distribution of Alzheimer disease and vascular pathology in brain aging.* Stroke, 2009. **40**(3): p. 983-986.
4. Calderón-Garcidueñas, L., et al., *Quadruple abnormal protein aggregates in brainstem pathology and exogenous metal-rich magnetic nanoparticles. The substantia nigrae is a very early target in young urbanites and the gastrointestinal tract likely a key brainstem portal.* Environmental Research, 2020: p. 110139.
5. van der Weerd, L., et al., *Effects of Alzheimer's disease and formalin fixation on the different mineralised*-iron forms in *the human brain.* Scientific Reports, 2020. **10**(1): p. 16440.
6. Brem, F., et al., *Characterization of iron compounds in tumour tissue from temporal lobe epilepsy patients using low temperature magnetic methods.* Biometals, 2005. **18**(2): p. 191-197.
7. Calderón-Garcidueñas, L., et al., *Combustion- and friction-derived magnetic air pollution nanoparticles in human hearts.* Environmental Research, 2019. **176**: p. 108567.
8. Dobson, J. and P. Grassi, *Magnetic properties of human hippocampal tissue—Evaluation of artefact and contamination sources.* Brain Research Bulletin, 1996. **39**(4): p. 255-259.
9. Hirt, A.M., et al., *Anomalous magnetic properties of brain tissue at low temperature: The 50 K anomaly.* Journal of Geophysical Research: Solid Earth, 2006. **111**(B12).
10. Kirschvink, J.L., A. Kobayashi-Kirschvink, and B.J. Woodford, *Magnetite biomineralization in the human brain.* Proceedings of the National Academy of Sciences, 1992. **89**(16): p. 7683-7687.
11. Maher, B.A., et al., *Magnetite pollution nanoparticles in the human brain.* Proceedings of the National Academy of Sciences, 2016. **113**(39): p. 10797-10801.
12. Sant'Ovaia, H., et al., *Magnetic susceptibility and isothermal remanent magnetization in human tissues: a study case.* Biometals, 2015. **28**(6): p. 951-8.
